# Irrigation fluid absorption induces metabolic and oxidative stress dysregulation activating NLRP3 and NF-κB pathways to drive hippocampal inflammatory responses

**DOI:** 10.3389/fphar.2026.1881021

**Published:** 2026-10-13

**Authors:** Qihui Zheng, Guilin Wang, Yutong Lu, Haitao Jia, Jiangtao Bai, Luyang Zhang, Ting Guo, Qi Jin, Zhilong Dong, Jun Mi, Yingbin Wang, Enguang Yang, Zhiping Wang

**Affiliations:** 1 Department of Anesthesiology, The Second Hospital of Lanzhou University, Lanzhou, China; 2 Department of Urology, The Second Hospital of Lanzhou University, Gansu Province Clinical Research Center for Urinary System Disease, Lanzhou, China

**Keywords:** hippocampal neuroinflammation, irrigation fluid absorption, metabolic stress, NF-κB pathway, NLRP3 inflammasome, oxidative stress

## Abstract

**Background:**

Irrigation fluid absorption during endoscopic procedures can lead to serious complications, but its effects on hippocampal function remain poorly understood. This study aimed to investigate the impact of irrigation fluid absorption on hippocampal metabolic homeostasis and inflammatory responses.

**Methods:**

Adult female Sprague-Dawley rats received intravenous 0.9% saline (control, 40 mL/kg, or 80 mL/kg) to simulate irrigation fluid absorption. Saline was infused over 30 min, and the animals were euthanized 15 min after cessation of anesthesia, corresponding to 45 min after initiation of the infusion. We assessed emergence time from anesthesia, hippocampal energy metabolism through targeted metabolomics, mitochondrial ultrastructure via transmission electron microscopy (TEM), oxidative stress markers, and NLRP3- and NF-κB-related inflammatory markers.

**Results:**

The 80 mL/kg group showed significantly prolonged anesthesia emergence time compared to controls (59.0 ± 13.5 vs. 31.7 ± 4.5 s; p < 0.01). Metabolomics revealed dose-dependent perturbations in the TCA cycle, glycolysis, and oxidative phosphorylation (p < 0.05). TEM analysis demonstrated severe mitochondrial ultrastructural damage in hippocampal neurons, including dose-dependent swelling, internal membrane disruption, and matrix dissolution. Both absorption groups showed altered glutathione redox status, characterized by increased GSSG levels and decreased GSH/GSSG ratios (p < 0.01), whereas SOD, CAT, MDA, NADPH, and GSH exhibited heterogeneous and non-linear changes across doses. At this early endpoint, increased NLRP3, P50/P65, IL-6, IL-18, and IL-33 immunoreactivity, together with decreased IκB immunoreactivity, indicated early changes in NLRP3- and NF-κB-related inflammatory signaling.

**Conclusion:**

Excessive irrigation fluid absorption induces hippocampal neuroinflammation through metabolic disturbance and redox dysregulation, which activates NLRP3 and NF-κB pathways. These findings suggest potential therapeutic targets for preventing neurological complications during endoscopic procedures.

## 1 Introduction

Numerous endoscopic procedures, including transurethral resection of the prostate (TURP), hysteroscopic surgery, and percutaneous nephrolithotomy, rely on irrigation fluids to maintain clear surgical fields and remove debris and blood. A significant complication of these interventions is the systemic absorption of irrigation fluids (Ortner et al., 2021), which primarily occurs due to elevated irrigation pressure that forces the fluid into systemic circulation through exposed vascular sinuses and damaged adjacent tissues (Garry et al., 1992; Gray et al., 2001; Zhang et al., 2024).

During TURP procedures, extensive absorption of irrigation fluids may lead to transurethral resection (TUR) syndrome, characterized by manifestations ranging from fluid overload to neurological impairments. Similar symptoms may be observed in other endoscopic interventions with excessive fluid absorption (Ortner et al., 2021). A prospective multicenter study of 10,654 patients undergoing TURP in 2002–2003 reported a TUR syndrome incidence of 1.4% (Reich et al., 2008). Although large-volume absorption events are relatively rare, they can exceed 3 L in certain patients and necessitate intensive medical care due to complications including pulmonary edema, congestive heart failure, hyponatremia, and hypothermia (Park, 2019). In particular, hyponatremia may cause cerebral edema and neurological symptoms such as confusion, seizures, and coma (Okazaki et al., 2020; Murt and Kalantar-Zadeh, 2023). Moreover, certain irrigation fluid constituents, such as glycine, possess direct neurotoxic potential on the brain and retina, potentially leading to visual impairments and altered evoked potentials (Mantha et al., 1991; Karci and Erkin, 2003). While the use of physiological saline reduces risks of hemolysis, hyponatremia, and glycine toxicity by preserving postoperative serum sodium levels (Hahn, 1991), isotonic saline overload induces significant plasma expansion confined to the extracellular compartment—without triggering osmotic diuresis. Notably, a randomized controlled trial has shown that intravenous administration of 30 mL/kg of 0.9% saline can provoke interstitial pulmonary edema in healthy volunteers (Li et al., 2021).

The hippocampus plays a critical role in memory formation, learning, emotional regulation, and spatial navigation (Strange et al., 2014; Lisman et al., 2017). However, confusion, seizures, and coma are not specific to hippocampal dysfunction. The incidence of hippocampal dysfunction specifically attributable to irrigation-fluid absorption remains undetermined. Experimental studies have shown that disrupting lactate transport in the rat hippocampus impairs long-term memory (Suzuki et al., 2011). Peripheral surgical trauma has also been shown to elicit hippocampal inflammation and memory deficits in mice (Feng et al., 2017). These findings support the hippocampus as a biologically relevant candidate region for examining metabolic and inflammatory responses to systemic perioperative disturbances. Whether acute systemic saline loading alters hippocampal metabolic homeostasis and inflammatory responses therefore warrants experimental investigation. We hypothesize that significant fluid absorption may cause hippocampal injury. This study aims to elucidate the connection between irrigation fluid absorption and hippocampal injury, thereby addressing an important gap in the existing literature.

## 2 Materials and methods

### 2.1 Animal procedures

Eight-week-old female Sprague-Dawley rats were used in this study. A single-sex design was adopted in this exploratory study to avoid introducing sex as an additional biological factor in the relatively small experimental cohort. The use of female rats is also relevant to hysteroscopic procedures, in which systemic absorption of irrigation fluid may occur. However, female rats were not selected based on the assumption that they are more susceptible to osmotic disturbances or hippocampal injury following acute saline loading. A total of 18 rats were included and randomly assigned to the control, 40 mL/kg, and 80 mL/kg groups (n = 6 animals per group). The body weights of rats in all three groups ranged from 260 to 280 g, with no significant difference in mean body weight among the groups. All rats were housed in a specific-pathogen-free (SPF) animal facility. Rats were anesthetized with isoflurane. Anesthesia was induced by placing the rats in a transparent induction chamber filled with isoflurane. Following induction, anesthesia was maintained using a nose cone delivering 2%–3% isoflurane. To model acute systemic saline loading associated with irrigation-fluid absorption during endoscopic procedures, 40 mL/kg or 80 mL/kg of 0.9% saline was administered intravenously. The control group underwent the same anesthesia and experimental handling as the saline-treated groups but did not receive intravenous saline infusion. Immediately after induction, saline was infused intravenously at a steady rate for 30 min, and the anesthetic gas was discontinued upon completion of the infusion. The rats were euthanized by cervical dislocation 15 min after cessation of anesthesia. Immediately after euthanasia, the brain was rapidly removed and placed on ice. The cerebral hemispheres were separated along the midline, and the bilateral hippocampi were carefully dissected using standard anatomical landmarks. Thus, tissue collection occurred 45 min after initiation of the 30-min saline infusion. This endpoint was selected to capture immediate metabolic, oxidative, and inflammatory signaling responses to acute saline loading. The study was not designed to characterize the complete temporal evolution, peak, or persistence of hippocampal neuroinflammation. The time to emergence from anesthesia was recorded. (Post-anesthetic arousal in rats was defined as the return of standing, ambulation, exploratory behavior, and normal foraging and drinking behaviors.) All experimental procedures adhered to the National Institutes of Health Guide for the Care and Use of Laboratory Animals and were approved by the Ethics Committee of The Second Hospital of Lanzhou University (D2023-3).

### 2.2 Targeted energy metabolomics analysis

A predefined panel of 80 metabolites related to energy metabolism was analyzed using targeted metabolomics. HPLC-grade solvents (Merck, Sigma-Aldrich) were used for metabolite extraction. Hippocampal tissues were homogenized in 70% methanol, followed by protein precipitation and centrifugation (12,000 rpm, 4 °C), and the resulting supernatants were analyzed via LC-ESI-MS/MS (Waters ACQUITY UPLC BEH Amide column; QTRAP 6500+ system). Chromatographic separation employed a 15-min gradient (95%–50% acetonitrile). Targeted metabolites were quantified using multiple reaction monitoring (MRM) with optimized declustering potentials (DP) and collision energies (CE) under optimized ESI conditions (±5500 V, 550 °C). Extraction efficiency was validated through MRM parameter optimization and standard metabolite recovery tests. Multivariate analyses (PCA, OPLS-DA) and KEGG pathway enrichment were performed in R with unit variance scaling and permutation validation (n = 200). KEGG pathway significance was determined by hypergeometric test p-values. Pathway significance was defined as p < 0.05, and FDR correction was applied to account for multiple comparisons.

### 2.3 Analysis of SOD, CAT, NADPH, and NADP + levels

Levels of superoxide dismutase (SOD), catalase (CAT), nicotinamide adenine dinucleotide phosphate (NADPH), and NADP+ were determined using the enzyme-linked immunosorbent assay (ELISA) double-antibody sandwich method with 3,3′,5,5′-Tetramethylbenzidine (TMB) colorimetric detection. After tissue processing, pre-cooled phosphate-buffered saline (PBS) (0.01 M, pH 7.4) was added for homogenization. Centrifugation parameters were standardized across assays (10,000 × g, 5 min, 4 °C), and the supernatant was collected for analysis. SOD levels in the supernatant were measured using a rat SOD assay kit (Cat: RA20004, Bioswamp®, China). CAT levels were determined using a rat CAT assay kit (Cat: RA20631, Bioswamp®, China). NADP+/NADPH levels were assessed using corresponding assay kits from Jiangsu Jingmei Biotechnology Co., Ltd. (China). Target molecules in the samples bound to pre-coated antibodies and subsequently to biotin-labeled antibodies, forming antigen-antibody-enzyme complexes. The chromogenic substrate TMB turned blue under horseradish peroxidase enzyme catalysis and then yellow after acidification. Optical density was measured at 450 nm. The concentrations of SOD, CAT, NADP+, and NADPH in each sample were calculated using standard curves.

### 2.4 GSH/GSSG level determination

Glutathione (GSH) and glutathione disulfide (GSSG) levels were measured using a glutathione reduced/oxidized (GSH/GSSG) detection kit (Cat: BTK042, Bioswamp®, China). GSSG was reduced to GSH by glutathione reductase and reacted with the chromogenic substrate DTNB to produce yellow TNB. Total glutathione (GSH + GSSG) content was determined by measuring absorbance at 412 nm. GSSG content in samples was measured after eliminating GSH, with samples processed through rapid freezing in liquid nitrogen, treatment with a protein removal reagent, and appropriate dilution.

### 2.5 MDA level determination

Lipid peroxidation was assessed by measuring malondialdehyde (MDA) content using an MDA assay kit (Cat: BTK022, Bioswamp®, China). This kit utilizes the reaction between thiobarbituric acid (TBA) and MDA to produce a red product (MDA-TBA adduct). The MDA-TBA adduct shows maximum absorption at 535 nm, enabling colorimetric detection. After sample homogenization, MDA detection working solution was added, followed by heating in boiling water for 15 min. Samples were then cooled and centrifuged to collect the supernatant. Absorbance was measured at 532 nm. The amount of MDA-TBA adduct formed is proportional to the MDA content in the samples.

### 2.6 Immunohistochemistry (IHC)

Rat hippocampal tissues were fixed in formalin, embedded in paraffin, and sectioned at a thickness of 4 μm. For IHC, sections were incubated overnight at 4 °C with antibodies against Inhibitory κB (IκB), Interleukin-1 Beta (IL-1β), IL-6, IL-18, IL-33, NLR Family Pyrin Domain-Containing 3 (NLRP3), P50, P65, and Tumor Necrosis Factor-alpha (TNF-α), followed by detection using a two-step universal kit (PV-8000–1). The hippocampus was dissected and processed as an intact region and was not physically subdivided into dorsal and ventral components before histological preparation. This whole-hippocampus approach was selected because the primary objective of the study was to characterize the overall acute metabolic, oxidative, and inflammatory response of the hippocampus rather than functional specialization along its longitudinal axis. Accordingly, the present analysis does not permit separate inference regarding dorsal versus ventral hippocampal responses. For each animal, three anatomically matched coronal hippocampal sections separated by approximately 100 μm were analyzed. Three non-overlapping fields were acquired from the same predefined hippocampal region in each section at the same magnification, using identical illumination, exposure, and white-balance settings. All images were coded before analysis and quantified by an investigator blinded to group allocation. DAB staining was separated from hematoxylin counterstaining using the Color Deconvolution function in ImageJ. A uniform threshold was applied to all images for each marker, and immunoreactivity was semi-quantified as integrated optical density normalized to the analyzed area. Measurements from the nine fields obtained from each animal were averaged to generate one biological value per animal.

### 2.7 Transmission electron microscopy (TEM)

Transmission electron microscopy was performed using a JEM-1400FLASH microscope operated at 80 kV. Hippocampal tissue blocks were fixed in 2.5% glutaraldehyde for 24 h, washed with 0.1 M PBS, and post-fixed in 1% osmium tetroxide for 2 h. Samples were subsequently dehydrated through a graded ethanol series and processed for TEM according to standard ultrastructural preparation procedures. Mitochondrial ultrastructure was examined at ×8,000 and ×25,000 magnification. Morphological evaluation included mitochondrial shape and swelling, integrity of the outer and inner membranes/cristae, matrix density or rarefaction, and the presence of vacuolar degeneration or structural dissolution. TEM was selected because it enables direct visualization of mitochondrial ultrastructural integrity and therefore provides morphological evidence complementary to the metabolomic and redox measurements.

### 2.8 Statistical analysis

Measurement data are expressed as mean ± SD. Normality and homogeneity of variance were assessed using the Shapiro–Wilk test and Brown–Forsythe test, respectively, before parametric analyses. Student’s t-test was used for pairwise comparisons of individual metabolite levels between each saline-treated group and the control group, whereas one-way ANOVA was used to compare emergence time, oxidative/redox-related biochemical indices, and IHC measurements among the three experimental groups. Statistical analyses were performed using GraphPad Prism 9.5. P values < 0.05 were considered statistically significant.

## 3 Results

### 3.1 Fluid absorption leads to prolonged emergence time from anesthesia

The mean emergence times from anesthesia for the three groups were as follows: 31.67 (SD = 4.51) seconds for the control group, 37.67 (SD = 6.50) seconds for the 40 mL/kg saline group, and 59.00 (SD = 13.54) seconds for the 80 mL/kg saline group (Table 1). Statistical analysis revealed a significant difference in emergence times between the control group and the 80 mL/kg group (p < 0.01). However, no significant difference was observed between the control group and the 40 mL/kg group. These findings suggest that increased irrigation fluid absorption delays anesthesia recovery, particularly at higher volumes (80 mL/kg). However, emergence time does not specifically reflect hippocampal function.

**TABLE 1:** Emergence time from anesthesia.

Group	Mean emergence time (seconds)	Standard Deviation (seconds)	p-value
Control	31.67	4.51	​
40 mL/kg	37.67	6.50	p^a^ > 0.05
80 mL/kg	59.00	13.54	p^b^ < 0.01

^a^ 40 mL/kg vs. Control.

^b^ 80 mL/kg vs. Control. n = 6 animals per group.

### 3.2 Irrigation fluid absorption alters hippocampal energy metabolism

Changes in cellular energy metabolism critically affect biological functions. The hippocampus plays a crucial role in cognitive functions and the recovery process following anesthesia. To investigate whether alterations in the central nervous system induced by irrigation fluid absorption are mediated through energy metabolism, targeted metabolomics analysis was conducted on hippocampal tissues from the three groups. The results demonstrated distinct clustering patterns of energy metabolites among the groups. While the principal component analysis (PCA) plot (Figure 1a) showed some degree of clustering, the separation between the high-dose (80 mL/kg) and control (CK) groups was not prominent. This may reflect compensatory responses or inter-individual variability under high-volume absorption conditions. In contrast, supervised partial least squares discriminant analysis (PLS-DA) demonstrated clearer separation between both the 40 mL/kg vs. CK and 80 mL/kg vs. CK comparisons (Figures 1b,c), suggesting that absorption volume alters metabolomic signatures. The heatmap (Figure 1f) showed intensity variations in several metabolites between the fluid absorption groups and controls. KEGG pathway enrichment analysis, based on significantly altered metabolites, revealed disturbances in critical pathways such as the tricarboxylic acid (TCA) cycle, glycolysis, and oxidative phosphorylation (Figures 1d,e), with more pronounced changes in the 80 mL/kg group. These findings suggest that irrigation fluid absorption disrupts hippocampal energy metabolism, particularly under the high absorption condition (80 mL/kg).

**FIGURE 1:**
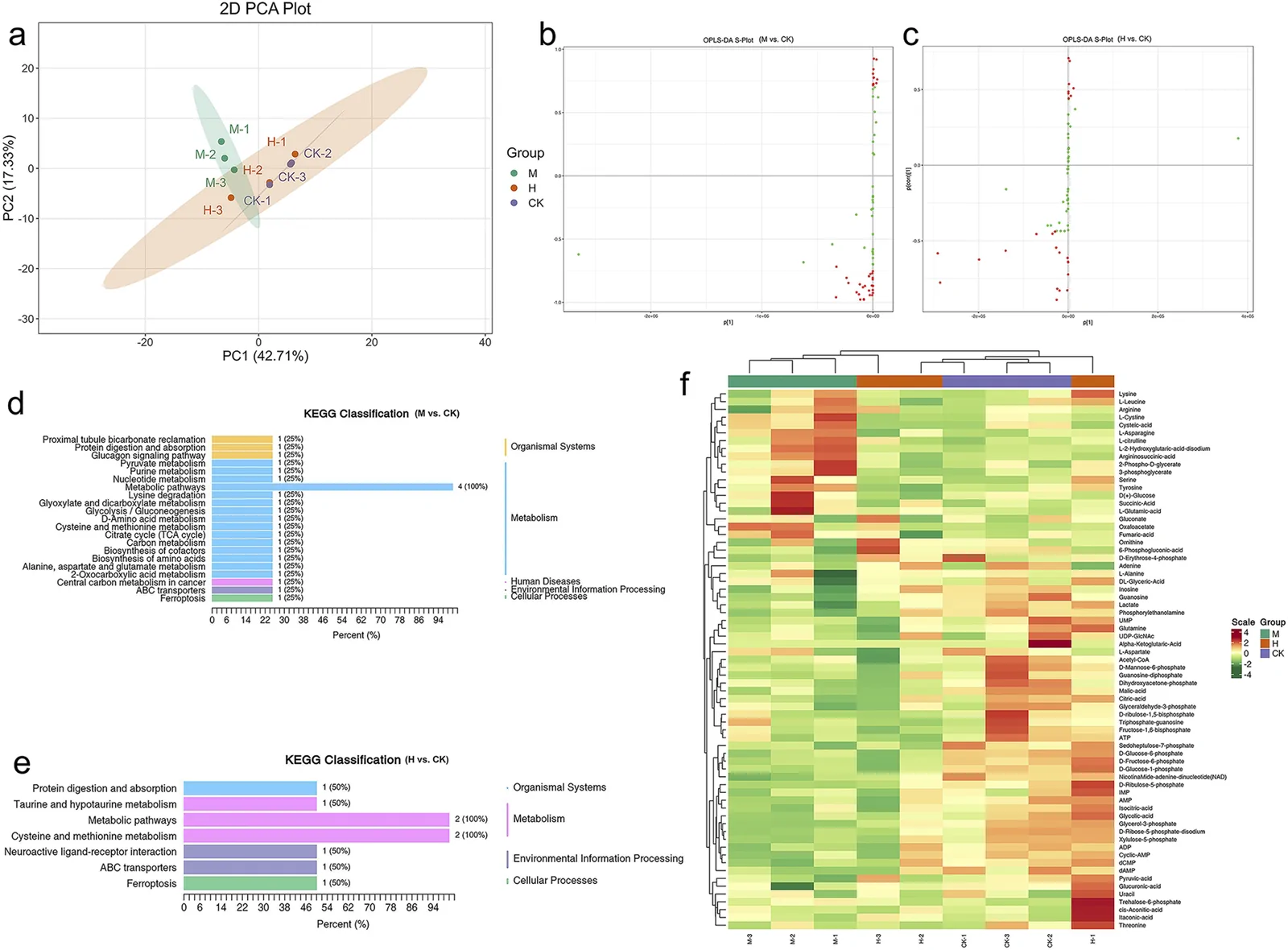
Effects of irrigation fluid absorption on hippocampal energy metabolism. Group definitions: M (Moderate absorption: 40 mL/kg), H (High absorption: 80 mL/kg), CK (Control: 0 mL/kg). **(a)** PCA of energy metabolites in the M, H, and CK groups; **(b)** PLS-DA between M and CK; **(c)** PLS-DA between H and CK; **(d)** Metabolic pathway enrichment analysis for M vs. CK comparison; **(e)** Metabolic pathway enrichment analysis for H vs. CK comparison; **(f)** Heatmap of differential metabolites across all groups. n = 3 animals per group.

### 3.3 Irrigation fluid absorption alters hippocampal mitochondrial morphology

Mitochondria serve as central hubs of cellular energy metabolism. We further investigated whether the metabolic alterations induced by irrigation fluid absorption occur through mitochondrial-mediated mechanisms. TEM observations (Figure 2) revealed significant ultrastructural changes in the mitochondria of hippocampal neurons in both the 40 mL/kg and 80 mL/kg groups compared to the control group. In the control group, the mitochondria exhibited a regular oval shape, clear internal membrane structure, and a dense matrix. In the 40 mL/kg group, some mitochondria displayed swelling and disruption, with blurred internal membrane structures. In contrast, the 80 mL/kg group demonstrated severe swelling and deformation, featuring unclear internal membrane structures, dissolved matrices, and the appearance of vacuolar changes. Additionally, some mitochondria in the 80 mL/kg group were completely dissolved, leaving only residual membrane fragments. These findings suggest that excessive fluid absorption is associated with mitochondrial ultrastructural alterations in the hippocampus, including mitochondrial swelling, membrane disruption, and matrix dissolution, with the most pronounced changes observed in the 80 mL/kg group. These observations refer specifically to mitochondrial morphology and should not be interpreted as evidence of hippocampal tissue swelling or cerebral edema. These findings demonstrate acute mitochondrial ultrastructural alterations in hippocampal neurons, although their functional consequences remain undetermined.

**FIGURE 2:**
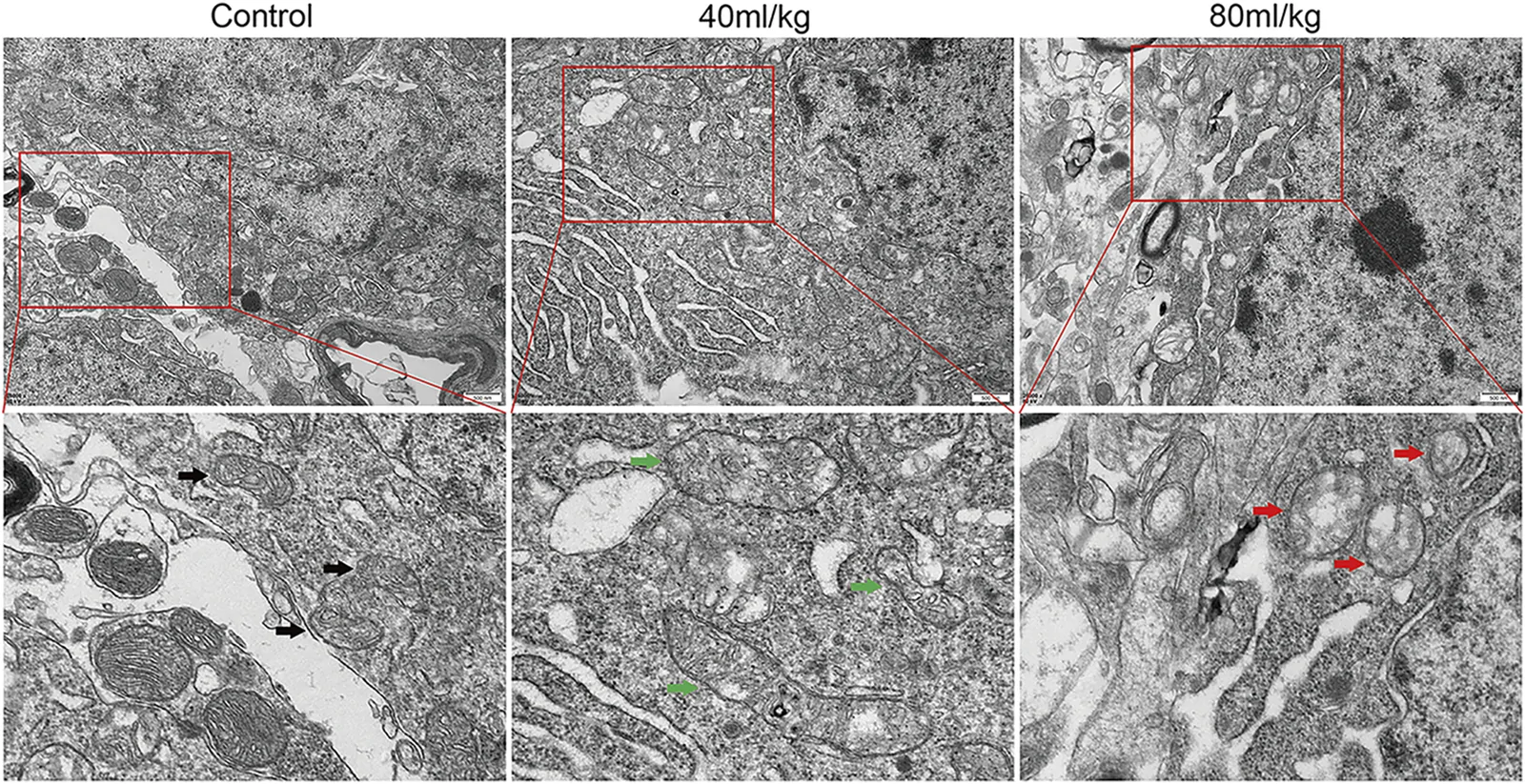
Transmission electron microscope images of hippocampal neuronal mitochondria in different groups. 25,000× magnification, 80 kV. Arrows indicate the corresponding mitochondrial ultrastructure. Scale bars are shown in each panel. n = 3 animals per group.

### 3.4 Irrigation fluid absorption affects hippocampal oxidative stress

Changes in mitochondrial homeostasis significantly influence cellular redox balance. We further examined oxidative stress and redox-related markers following irrigation fluid absorption. The results (Figure 3) indicated that SOD activity showed an increasing trend in the 40 mL/kg and 80 mL/kg groups; however, the differences were not statistically significant. CAT activity significantly decreased (p < 0.05) in the 40 mL/kg group but significantly increased (p < 0.05) in the 80 mL/kg group. MDA levels were slightly elevated in the 40 mL/kg group and showed a decreasing trend in the 80 mL/kg group, although inter-group differences were not statistically significant.

**FIGURE 3:**
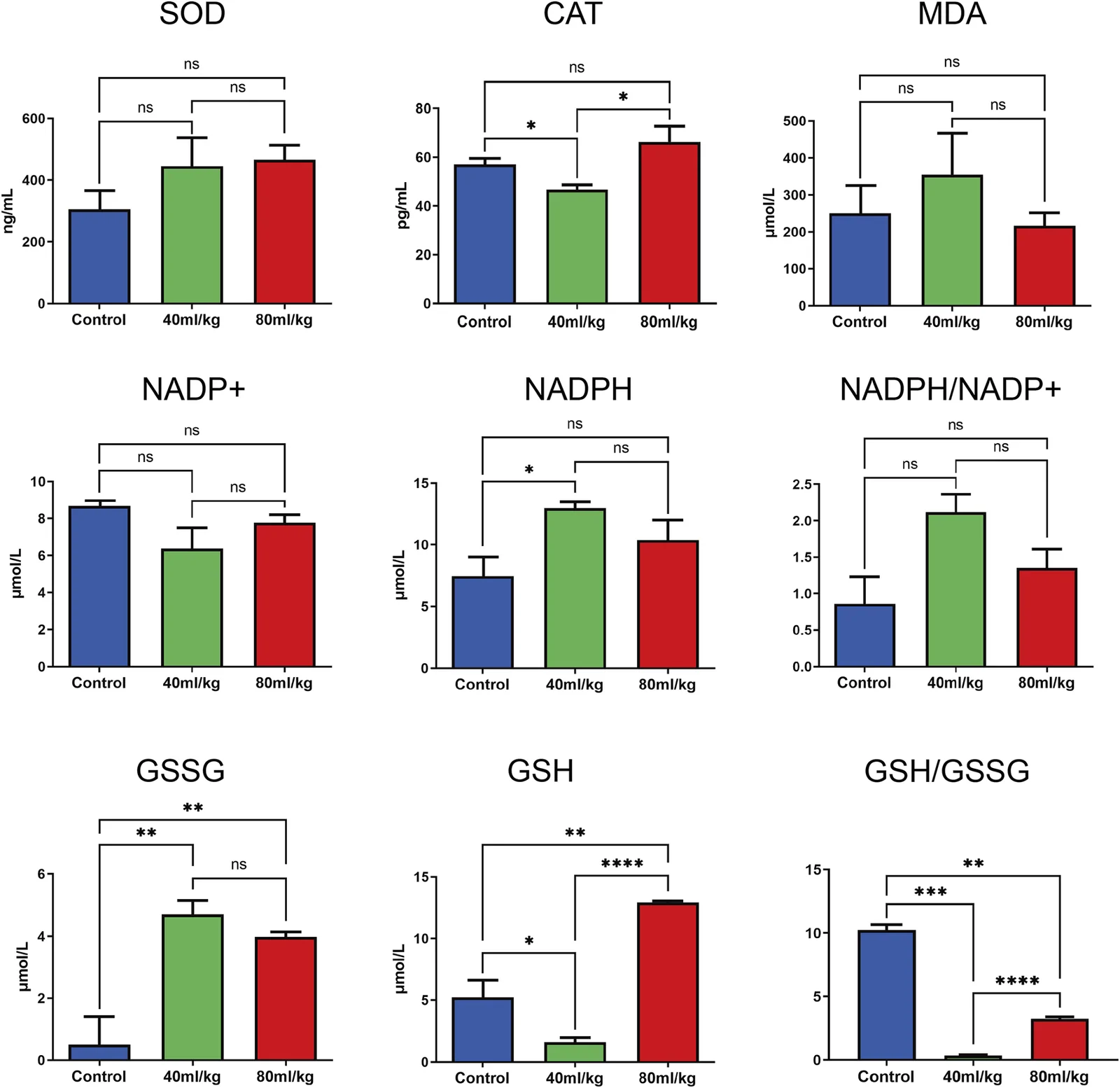
Analysis of oxidative stress markers and redox status parameters in different groups. The levels of antioxidant enzymes (SOD and CAT), lipid peroxidation marker (MDA), redox cofactors (NADP+/NADPH), and glutathione system components (GSH and GSSG) were measured. The ratios of NADPH/NADP+ and GSH/GSSG were calculated to assess redox status. Statistical significance is indicated as: ns = not significant; *p < 0.05; **p < 0.01; ***p < 0.001; ****p < 0.0001. n = 3 animals per group.

Regarding redox status, there was no statistically significant difference in NADP + levels in both the 40 mL/kg and 80 mL/kg groups compared to the control group. Conversely, NADPH levels significantly increased (p < 0.05) in the 40 mL/kg group but returned to near-control levels in the 80 mL/kg group. The NADPH/NADP + ratio exhibited a trend similar to that of NADPH.

The glutathione system showed significant changes. Compared to the control group, the levels of GSSG in both the 40 mL/kg and 80 mL/kg groups were significantly elevated (p < 0.01). The GSH content in the 40 mL/kg group was significantly decreased (p < 0.05), while it was significantly increased in the 80 mL/kg group (p < 0.01). The GSH/GSSG ratios in both the 40 mL/kg and 80 mL/kg groups were significantly decreased (p < 0.01).

### 3.5 Metabolites and oxidative stress products as damage-associated molecular patterns (DAMPs) promote hippocampal inflammatory changes via NLRP3 and NF-κB signaling pathways

Aberrant cellular metabolites and redox products may act as DAMPs that initiate inflammatory responses. The NLRP3 inflammasome serves as a receptor for multiple oxidative stress products, activating the expression of downstream inflammatory cytokines, including IL-6, IL-1β, IL-18, and IL-33. IHC results (Figure 4) demonstrated that NLRP3 immunoreactivity significantly increased with increasing fluid volume (p < 0.01), while levels of pro-inflammatory cytokines IL-6, IL-18, and IL-33 exhibited a marked upward trend, suggesting early NLRP3-related inflammatory signaling. Similarly, compared to the control group, the 80 mL/kg fluid absorption group showed significantly elevated TNF-α levels (p < 0.01). TNF-α can activate the NF-κB signaling pathway to promote the expression of inflammatory mediators. Further analysis revealed that IκB expression levels significantly decreased with increasing fluid volume, while the expression levels of NF-κB signaling pathway proteins P50 and P65 were markedly elevated, consistent with early NF-κB-related signaling changes. These results indicate that excessive fluid absorption is associated with early changes in NLRP3- and NF-κB-related inflammatory markers. However, the single early endpoint does not establish the complete temporal evolution of these inflammatory responses.

**FIGURE 4:**
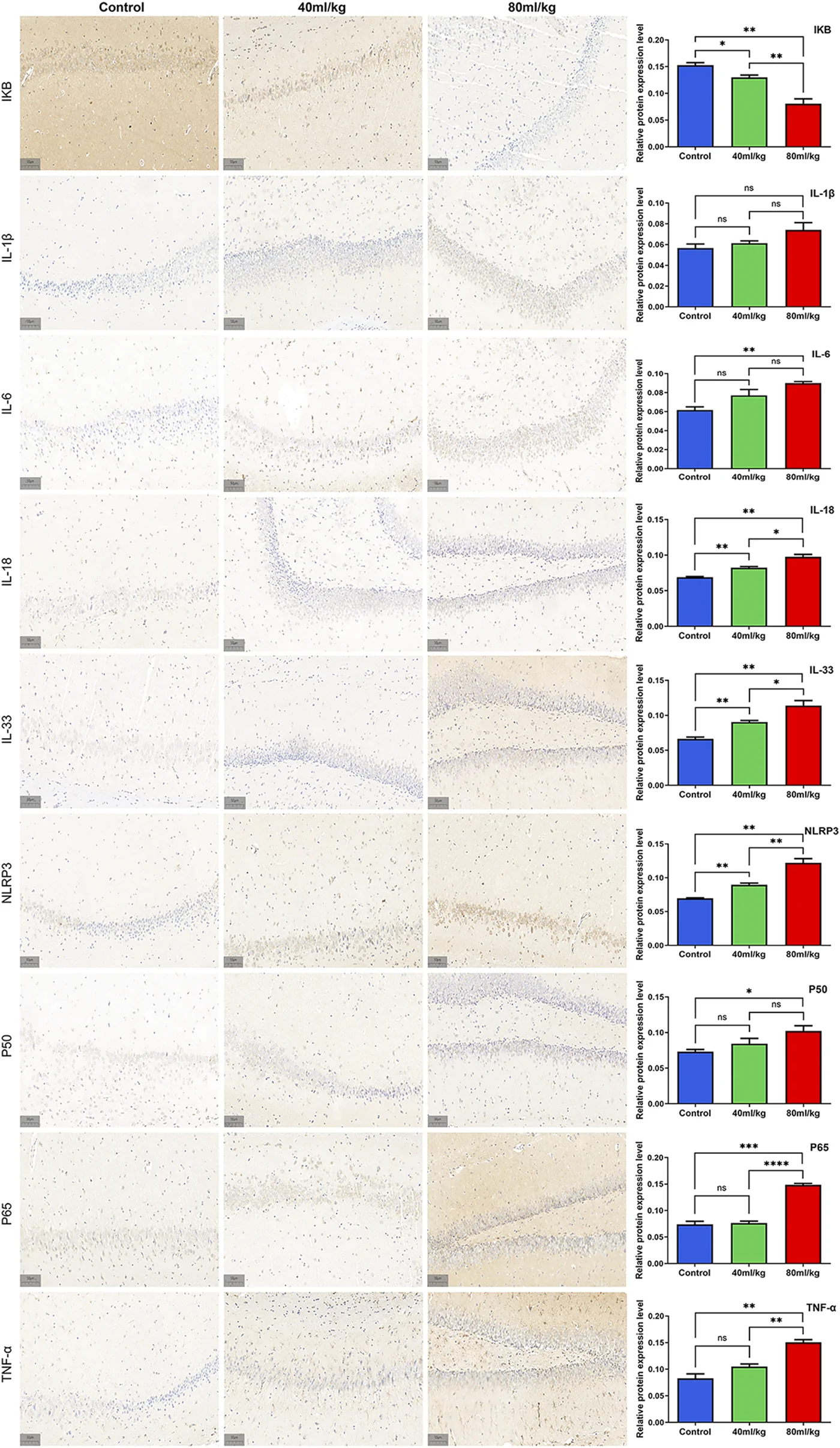
Immunohistochemical staining of inflammatory factors in hippocampal tissue from different groups. Immunohistochemistry staining shows the expression of IκB, IL-1β, IL-6, IL-18, IL-33, NLRP3, P50, P65, and TNF-α in hippocampal tissue of the control group, 40 mL/kg fluid absorption group, and 80 mL/kg fluid absorption group (scale bar = 50 μm). Bar graphs show the relative expression levels of each protein. Data are presented as mean ± SD. *p < 0.05, **p < 0.01, ***p < 0.001, ****p < 0.0001; ns: not significant. n = 3 animals per group.

## 4 Discussion

Our study provides critical insights into the neurological consequences of excessive irrigation fluid absorption by establishing a direct link between fluid overload, hippocampal metabolic/oxidative stress dysregulation, and NLRP3/NF-κB-mediated neuroinflammation (Figure 5). While isotonic saline is widely used to mitigate dilutional hyponatremia and glycine toxicity in endoscopic surgeries (Hahn, 1991; Yousef et al., 2010; Ewees et al., 2019), our findings highlight its underappreciated risks: systemic absorption of isotonic saline disrupts hippocampal homeostasis through interrelated mechanisms involving energy metabolism impairment, mitochondrial damage, and redox-inflammatory cascades.

**FIGURE 5:**
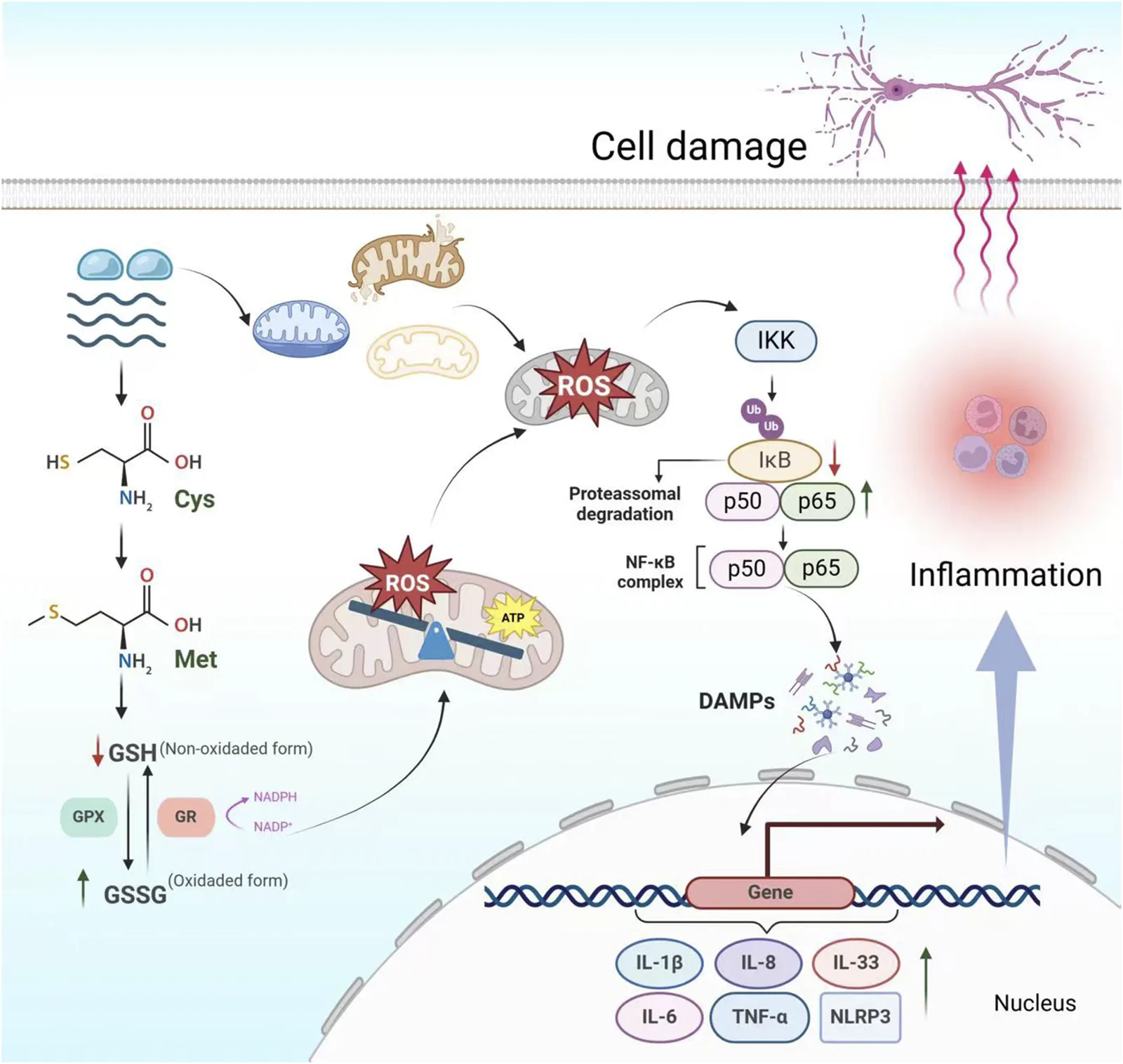
Mechanistic overview of hippocampal neuroinflammation induced by irrigation fluid absorption during endoscopic procedures. Fluid overload disrupts cellular homeostasis, triggering mitochondrial membrane potential changes and subsequent ROS production. The resulting metabolic and oxidative stress alters redox balance, promoting cysteine-to-methionine conversion and decreasing the GSH/GSSG ratio while activating antioxidant systems (GPX/GR with NADPH cofactor). Oxidative stress products function as DAMPs, activating the NF-κB pathway through IKK-mediated IκB phosphorylation and proteasomal degradation, leading to p50/p65 heterodimer nuclear translocation. Concurrently, mitochondrial ROS and released ATP trigger NLRP3 inflammasome assembly and activation. These pathways synergistically induce pro-inflammatory cytokine expression (IL-1β, IL-6, IL-8, TNF-α, IL-33) and NLRP3 upregulation, promoting neuroinflammation and subsequent hippocampal neuronal damage, which may underlie post-operative cognitive dysfunction following endoscopic procedures with significant irrigation fluid absorption.

The prolonged emergence time observed following high-volume saline loading may reflect an acute systemic effect of excessive fluid absorption. This is consistent with clinical observations of delayed neurological recovery in TUR syndrome (Okazaki et al., 2020; Ortner et al., 2021; Murt and Kalantar-Zadeh, 2023). However, emergence time reflects global recovery from anesthesia and cannot be specifically attributed to hippocampal dysfunction. Because hippocampal-dependent behavioral assessments, such as the Morris water maze, novel object recognition, or Y-maze, were not performed, the present study cannot determine whether the observed tissue-level alterations result in impaired learning, memory, or cognition. Future studies incorporating hippocampal-dependent behavioral assessments will be necessary to determine whether these acute molecular and ultrastructural alterations translate into functional cognitive consequences. The absence of significant differences between the 40 mL/kg group and the control group may be attributed to the body’s ability to maintain homeostasis through protective regulation and compensatory mechanisms during small-volume fluid absorption.

The observed metabolic and mitochondrial alterations suggest that acute saline loading may disturb neuronal energy homeostasis. Because mitochondrial integrity is closely linked to ATP production and cellular metabolic stability, structural mitochondrial alterations may contribute to the metabolic disturbances observed in the hippocampus. Such mitochondrial dysfunction parallels neurodegenerative pathologies (Odnokoz et al., 2021; Percio et al., 2024) and may arise from fluid-induced edema (Michinaga and Koyama, 2015), electrolyte shifts (Tombaugh and Somjen, 1996; Funes and de Morais, 2017), and altered pH (Imenez Silva et al., 2021), which collectively destabilize the hippocampal microenvironment. The metabolic responses did not consistently follow a linear dose-dependent pattern, suggesting that acute saline loading may trigger dynamic and potentially compensatory metabolic responses. Taken together, these findings suggest that irrigation fluid absorption—particularly at higher volumes—perturbs hippocampal energy metabolism, although the response patterns may not be linear due to dynamic regulatory mechanisms. We acknowledge the complexity of these results and will further refine experimental design and increase sample size in future studies to explore these patterns in greater detail. The interplay between mitochondrial alterations and redox imbalance may contribute to neuronal injury. The heterogeneous redox responses observed in the present study may reflect different phases or magnitudes of acute compensatory antioxidant responses, although this interpretation remains speculative. Alterations in glutathione homeostasis and antioxidant responses have also been described in mitochondrial and neurodegenerative disorders (Enns et al., 2014; Wei et al., 2020; Pisoschi et al., 2021). Mitochondrial dysfunction may contribute to redox imbalance through increased electron leakage and ROS generation (Enns et al., 2014; Percio et al., 2024), but ROS production was not directly measured in the present study. Therefore, our findings support acute redox dysregulation, particularly involving the glutathione system, rather than a uniform, dose-dependent, or robust oxidative injury response. Because only a single acute endpoint was examined, the magnitude, temporal progression, and causal role of these redox alterations cannot be definitively determined. Further studies incorporating direct ROS measurements, multiple sampling time points, and antioxidant interventions are required to clarify the underlying mechanisms.

The inflammatory response in the hippocampal tissue caused by fluid absorption may be related to the release of DAMPs. DAMPs are endogenous molecules produced by cells or tissues that are released through mechanisms such as membrane rupture, necrosis, and active secretion upon cellular damage, triggering various biological responses like inflammation, proliferation, and tissue injury (Murao et al., 2021; Nakano et al., 2022). The release and oxidation of mitochondrial DAMPs can activate pattern recognition receptors, alter cellular metabolism, enhance oxidative stress, and promote systemic inflammatory responses (Koenig and Buskiewicz-Koenig, 2022). The inflammatory profile observed in the present study is consistent with early involvement of NLRP3- and NF-κB-related signaling. Our findings align with neurodegenerative disease models where NLRP3 activation exacerbates central nervous system injury (O’Brien et al., 2020; Feng et al., 2021), suggesting shared pathways between surgical complications and chronic neurodegeneration. We speculate that the release of damage-associated molecular patterns may contribute to the activation of NLRP3- and NF-κB-related inflammatory pathways. The concurrent changes in NF-κB-related markers are compatible with involvement of this pathway, although the present measurements do not establish a complete signaling sequence. However, it should be noted that the priming signal for NLRP3 activation in this study remains unclear, and TNF-α may be a potential priming signal (Yang et al., 2025). Furthermore, ROS, known activators of NLRP3, might be involved in NLRP3 activation. Importantly, changes in inflammatory cytokine immunoreactivity alone do not establish NLRP3 inflammasome activation. Canonical NLRP3 inflammasome activation involves caspase-1-mediated processing of pro-inflammatory cytokines such as IL-1β and IL-18; however, these activation-specific events were not directly measured in the present study. Similarly, an increase in p65 expression alone is insufficient to prove NF-κB activation–phosphorylation of p65 is the usual indicator, but a reduction in IκB can serve as evidence for its activation. Although rapid NF-κB responses can occur within minutes in hippocampal neurons (Schwamborn et al., 2017), hippocampal tissue in the present study was collected at a single early time point—45 min after infusion initiation—and the findings should therefore be interpreted as early pathway-associated changes rather than a complete characterization of NF-κB and NLRP3 activation. Additional time-course studies at 1, 6, and 24 h are required to determine the onset, peak, and persistence of these inflammatory responses.

Another limitation is that only female rats were included in the present study, and estrous stage and circulating estrogen concentrations were not assessed. Therefore, the present data cannot determine whether sex or ovarian hormonal status modulates the hippocampal responses to acute saline loading. Future studies incorporating both sexes and predefined assessment of estrous status are needed to address this issue.

These findings carry significant clinical implications. First, intraoperative monitoring of absorption volumes could help identify patients at risk for hippocampal injury. Second, adjuvant therapies targeting oxidative stress (e.g., N-acetylcysteine) or neuroinflammation (e.g., NLRP3 inhibitors) might mitigate perioperative neural damage. Third, the observed dose-response relationship supports protocol modifications limiting maximum irrigation volumes. Implementing these strategies could improve cognitive outcomes in patients undergoing prolonged endoscopic procedures.

In conclusion, our study demonstrates that excessive irrigation fluid absorption can prolong emergence time from anesthesia and induce metabolic and oxidative stress dysregulation activating NLRP3 and NF-κB pathways to drive hippocampal inflammatory responses. The identification of NLRP3/NF-κB activation as central mediators provides potential therapeutic targets for preventing surgery-associated neural injury. Future investigations should explore: (1) temporal patterns of hippocampal damage progression, (2) blood-brain barrier permeability changes during fluid overload, and (3) neuroprotective efficacy of antioxidants in this context.

## Data Availability

The datasets presented in this study can be found in online repositories. The names of the repository/repositories and accession number(s) can be found below: https://osf.io/436b7/?view_only=94f5183ce4b34bef9fb80426225500e7 (OSF).
